# General practitioners’ perceived indicators of vulnerability in pregnancy- A qualitative interview study

**DOI:** 10.1186/s12875-021-01439-3

**Published:** 2021-06-26

**Authors:** L Brygger Venø, DE. Jarbøl, LB. Pedersen, J Søndergaard, RK Ertmann

**Affiliations:** 1 Research Unit of General Practice, Department of Public Health, University of Southern, Odense, Denmark; 2 DaCHE – Danish Centre for Health Economics, Department of Public Health, University of Southern, Odense, Denmark; 3 Research Unit of General Practice, Department of Public Health, University of Copenhagen, Copenhagen, Denmark

**Keywords:** Vulnerability, Pregnancy, Antenatal care, General practice

## Abstract

**Objective:**

To explore general practitioners’ (GPs’) perceived indicators of vulnerability among pregnant women in primary care.

**Design:**

A qualitative study with semi-structured in-depth focus group interviews.

**Setting:**

General practices located in a mixture of urban, semi-urban and rural practices throughout the Region of Southern Denmark

**Subjects:**

Twenty GPs.

**Main outcome measures:**

Through qualitative analysis with systematic text condensation of the interview data, the following themes emerged: (1) obvious indicators of vulnerability—i.e. somatic or psychological illnesses, or complex social problems and 2) intangible indicators of vulnerability – i.e. identification depended on the GPs’ gut-feeling.

From the GPs’ perspective, the concept of vulnerability in pregnancy were perceived as the net result of risk factors and available individual and social resources, with a psychosocial etiology as the dominant framework.

**Conclusions:**

The GPs demonstrated a broad variety of perceived indicators of vulnerability in pregnancy; most importantly, the GPs were aware of a group of pregnant women with intangible vulnerability mainly representing low resilience. Despite not fitting into the GPs' perceived concept of vulnerability, the GPs had a strong gut feeling that these women might be vulnerable. Misjudging the resources of pregnant women due to their physical appearance could delay the GPs’ identification of vulnerability. Future studies should explore the challenges GPs experiences when assessing vulnerability among pregnant women.

**Supplementary Information:**

The online version contains supplementary material available at 10.1186/s12875-021-01439-3.

## Introduction

Vulnerability among fertile women is increasing due to low psychosocial resources, such as social problems and mental health problems [1]. Undetected vulnerability during pregnancy may result in increased risk of complications either during the pregnancy, during the birth, or throughout childhood [2].

Vulnerability in pregnancy is described in the literature as psychosocial problems (i.e. history of anxiety or depression before or during pregnancy) or social problems (i.e. young age, lack of social support, being single, unemployed, low education level [3–8], history of adverse childhood experiences, poor socioeconomic status [9], stressful life events during pregnancy [6], or a history of domestic violence or abuse [10]). Additionally, many vulnerable women of fertile age, report alcohol consumption above the high risk level of seven units of alcohol per week [1, 11]. Finally, the capacity of the family, to withstand and rebound form stressful life challenges, named resilience, may affect the vulnerability [12].

In antenatal care, vulnerability constitutes a major factor in the development of inequalities in maternal and perinatal health [13, 14] and increases the risk of a debut or relapse of depression during pregnancy or a postpartum depression [3, 5, 8]. Additionally, vulnerability in pregnancy is significantly associated with negative birth outcomes, such as; preterm birth, low birth weight, low APGAR scores [15] and adverse outcomes in childhood i.e. disturbed mother–child relation with risk of child neglect, emotional problems and symptoms of attention deficit hyperactivity disorder [16–18]. Increased support in early pregnancy is crucial for vulnerable pregnant women which will help them benefit from the antenatal care system, and subsequently decrease related negative birth outcomes [19]. Furthermore, interventions for decreasing perinatal mental health problems have been shown to be cost-effective [20]. Therefore, vulnerable pregnant women are in need of extra support; during pregnancy, at birth and during the postpartum period [2]. However, very few vulnerable pregnant women proactively seek help [21] and are therefore reliant on the health professionals’ ability to determine vulnerability.

Over the past decades in Danish antenatal care all pregnant women are offered an early pregnancy consultation with their general practitioner (GP) between gestational week 6–10, with the purpose of evaluating the need for extra support during pregnancy by assessing the pregnant women's comorbid risks and psychosocial resources [2]. However, a report from the Danish National Board of Health indicated that it was challenging for the GPs to identify vulnerability among pregnant women, since only 25% of the most vulnerable pregnant women seen at the specialized social obstetric units were referred by their GP [22]. This finding is in line with challenges reported for GPs in the UK [23]. In order to identify a vulnerable pregnant woman, the GP must first understand which indicators implies vulnerability in pregnancy. Studies from the UK and Ireland have assessed GPs’ perceptions of perinatal mental health problems [24–26], however the GPs’ understanding of what indicates vulnerability in pregnancy was not explored.

As GPs, our pre-assumptions were formed through years of experience working with pregnant women and families in urban and rural settings and collaborating with social-obstetric outpatient units. We have experienced how vulnerable pregnant women were sometimes overlooked in pregnancy consultations and went through pregnancy without support, and some developed perinatal depression. Or pre-assumption was that different perceptions of indicators of vulnerability in pregnancy affected the GPs’ behavior when approaching the risk and resources of a pregnant women. The aim of this study is to explore GPs’ perceptions of what indicates vulnerability in pregnancy.

### Design, material, and methods

#### Design

This is a cross-sectional qualitative study based on semi-structured in-depth focus group discussions with GPs. It is part of a multi-method project exploring barriers and facilitators in assessing and in managing the antenatal care of vulnerable pregnant women from a user-perspective of the GPs. We chose the qualitative methodology to explore GPs perceived indicators of vulnerability in pregnancy. A qualitative design enabled us to explore the GPs understandings of and perceptions of indicators of vulnerability in pregnancy in the terms of “what, why and how”. The safe environment during interview encouraged them to disclose situations of deficient performances when identifying vulnerable pregnant women, and the dialogue rendered the GPs to reflect on their own practices.

We applied a pragmatic clinical empirical approach not driven by prior established theoretical framework. However, recognizing that our stance is always affected by theory, during process of analysis we searched for theories to support our data interpretation. We chose *the biopsychosocial model* by Engel [27] and *organismic thinking* by McWhinney [28] as a backdrop or inspiration.

The interview guide was developed by the research group, which consisted of four GPs (LBV, DEJ, RE, JS) and a questionnaire expert (LBP). Inspiration was gained from field observations conducted by LBV in social obstetric units which handled vulnerable pregnant women. The Consolidated criteria for Reporting of Qualitative research (COREQ) checklist was used to ensure transparency [29]. See the full checklist in Additional file 1.

#### Setting

The study was conducted in a general practice setting in the Region of Southern Denmark. In Denmark, general practices are organized as either single-handed practices (one GP per practice) or partnership practices varying in size (two-ten GPs per practice). The health care system in Denmark is tax funded and free of charge for the patient. Most Danes, both native and ethnic (99%) are registered with a GP of their own choosing, and the GP functions as a gatekeeper to secondary care [30].

Three antenatal care visits and one postnatal visit are offered by GPs. Danish antenatal care is a well-structured collaboration which usually involves GPs, midwives, health visitors (who are specialized municipal nurses) and the obstetric units. If the GP perceive a pregnant woman as vulnerable, the specialized social-obstetric units and municipal social workers can be involved depending on the severity of vulnerability [2]. During the first pregnancy consultation, the GP completes the mandatory pre-structured national pregnancy health record concerning; lifestyle habits (smoking, alcohol intake and use of addictive drugs), socioeconomic status, previous obstetric history and known somatic or psychological disorders. The national pregnancy health record helps the GP assess somatic and psychosocial vulnerability, which if detected, will require extra support during pregnancy. Like most services provided in general practice, the consultation is free of charge to patients. However, patients of ethnic origin who are not fluent in Danish and have lived in Denmark for longer than three years, are charged a fee for translator assistance [31].

### Data collection

#### Selection and recruitment

Twenty GPs from the Region of Southern Denmark participated in the study. The study aimed to recruit a purposive sample of GPs with respect to; gender, years of experience, practice type and various practice areas throughout the Region of Southern Denmark representing communities of all socio-economic layers. Respondents were recruited via letter, telephone, e-mail and snowball sampling. Almost 60 GPs were contacted, and the main reason for decline to participate in interviews was a high workload. Due to slow recruitment, the end sample consisted of a convenience sample representing only partnership practices. As GP trainees also conduct antenatal care consultations, we found it relevant to include GP trainees in the sample. Participant demographic details are shown in Table 1.

**Table 1:** Participant demographic details

Years of experience	Practice type	Practice area	Gender
0 years (GP trainees) (3)	Single-handed practices (0)	Urban area (5)	Female (12)
1–5 years (5)	Partnership practices (20)	Semi-urban area (11)	Male (8)
6–10 years (2)		Rural area (4)	
11–15 years (5)			
> 15 years (5)			

### Focus group discussions

Five focus group interviews with an average of four GPs were conducted by LBV and DEJ between March 2019 and January 2020. The interviews lasted approximately 60 min and took place at the research unit of general practice in Odense or in the local practice area of participating GPs. The first author LBV conducted the interviews with DEJ as an experienced moderator. LBV is a GP and PhD student who have attended courses on qualitative study design. DEJ, JS and RE are all senior researchers with experience of qualitative research traditions. Prior to interviews, the interviewer introduced the study aim; exploring the GPs experience working with vulnerable pregnant women, but without presenting our prior experiences and pre-assumptions in the field. The interviewer acted friendly, and like-minded among participants, ensuring a confident environment where all attitudes are equal and acceptable, and encouraging participants to share clinical examples—including when things were difficult. The interview guide provided a flexible frame with open-ended questions about the GPs’ perceptions of what indicates vulnerability in a pregnant woman and welcoming clinical examples. See the full interview guide in Additional file 2. Ongoing adjustments of the interview guide were made to elaborate on new perceptions. The first interview was a pilot with GPs working as part time researchers in our research unit, and therefore had a prior knowledge to the researcher team. Sampling ceased when new information stopped emerging, and data saturation was discussed among authors.

### Data management and analysis

All interviews were audio recorded and transcribed verbatim by LBV and uploaded to data processing software (NVivo) for coding and data organization. Open inductive coding with systematic text condensation [32] was used to ensure an in-depth investigation of themes and subthemes, where themes could freely emerge from data without being imposed by prior theory. Systematic text condensation is a method for thematic cross-case analysis which is inspired by phenomenological thinking and represents a pragmatic approach [32]. It consists of the following steps: 1) Total impression: gaining overview and elicit preliminary themes. 2) De-contextualization: developing code groups from preliminary themes, followed by identification and sorting of meaning bearing units containing information on our research question. 3) Condensation: sorting the meaning bearing units into subgroups exemplifying important aspects of every code group and condensing the content of subgroups into coherent text. 4) Synthesizing the condensed text from each subgroup into analytical text and resulting categories. Three authors (LBV, RE and DEJ) read the first two interviews. After the themes were discussed among the authors, LBV conducted the initial coding. The following stepwise analysis was conducted by LBV in cooperation with RE. The research team discussed and reflected on the findings until consensus was reached. Consistency were found between the data and the findings, as the findings were recontextualized against the original interview material. 

## Results

In general, the GPs perceived vulnerability as the net result of both risk factors and available individual and social resources. A psychosocial etiology appeared to be the dominant framework held by the GPs when conceptualizing vulnerability in pregnancy, as illustrated by one male GP when he said:

*“Vulnerability has to be understood in a social context and not only in an individual context”* (P18, male GP > 45 years old)

Factors of resilience inherent in the women and their available family supportive network was perceived important.

**Fig. 1:**
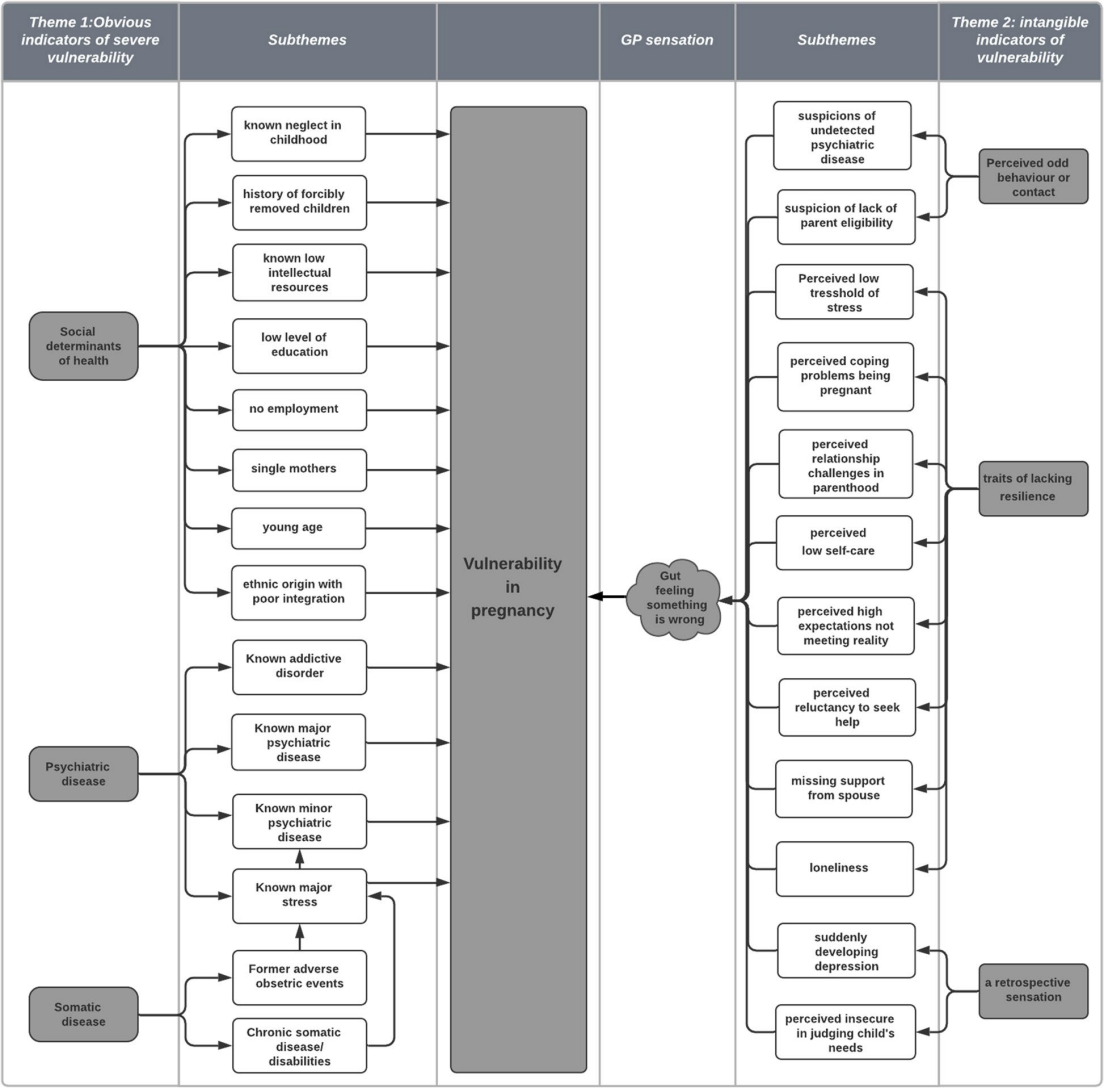
Overview of GPs perceived indicators of vulnerability in pregnancy, relating to their obviousness

As shown in Fig. 1, the GPs’ expressed a variety of conditions indicating vulnerability in pregnancy, originating from different comorbid conditions; somatic disease, psychiatric disease and social problems. Even though the GPs were not asked to classify indicators of vulnerability in pregnancy according to their apparent severity, the GPs’ perceived specific indicators as being more obvious and severe; whereas, other indicators were perceived as being intangible. Therefore, during the data analysis process of the GPs perceived indicators of vulnerability, to main themes emerged; 1) obvious indicators of severe vulnerability and 2) intangible indicators of vulnerability.

### The obvious indicators of vulnerability in pregnant women

The GP perceived that obvious indicators of vulnerability in pregnancy could be organized into three categories: social determinants of health, psychiatric diseases and somatic diseases.

*Social determinants of health* were complex social problems with known social cases in the system – such as known history of being neglected in childhood, history of having children forcibly removed or known low intellectual- or mental resources. Other social determinants of health were related to the level of socioeconomic status – i.e. low level of education or being unemployed. Also, sociodemographic factors as being a single pregnant woman was perceived an obvious indicator of vulnerability; especially if the woman was very young, with a broken relationship to the father or plans of parenting alone, and simultaneously having poor social resources. As one female GP said:

*“They’re young, haven’t known their partner for very long, have no education, no plans for their future and are often unemployed. We may know the family already, as a low social class family with low intellectual resources.”* (P14, female GP, > 45 years)

Poorly integrated women with an ethnic background were also perceived as vulnerable; since these women usually had a poor understanding of the language, the culture and local health system procedures. Lack of translator assistance made it difficult for the GPs to evaluate these women’s resources and guide them through the medical system. The GPs perceived that it was often necessary to refer them to the social-obstetric care units but experienced that the women’s poor economic and structural resources prevented them from attending the care units.

*Psychiatric diseases* indicating obvious vulnerability were known minor psychiatric disorders – i.e., attention deficit disorder or personality disorder(s)), major psychiatric disorder(s) – such as depression, anxiety or, schizophrenia, and known history of abuse of alcohol, drugs or addictive medicine. Whether the above pertained to the pregnant woman or her partner, they were perceived to indicate vulnerability.

*Somatic diseases* indicating obvious vulnerability could be history of severe obstetric complications or chronic somatic comorbidity due to the risk of complicating pregnancy. Additionally, presence of chronic somatic disease was perceived to increase the degree of vulnerability, since they naturally caused a higher level of stress from worries.

Finally, the addition of several indicators of vulnerability as psychiatric diseases concomitant with coping problems from disabilities or chronic diseases were perceived to increase the degree of vulnerability.

*“I had a patient with a hearing disability who was pregnant(...), however her real challenge is her many psychiatric challenges as she had been mentally unstable with poor self-care and difficulties managing social challenges(..) plus, I don’t think her intelligence level is very high”* (P15, female GP,< 45 years)

Conversely, some GPs reported being positively surprised by patients perceived as being obvious vulnerable. This was the case in situations where pregnant women, typically the young women or women with psychiatric disease(s), appearing with skills of resilience, growing with the task, and becoming brilliant mothers. However, mostly it demanded great social support from cross-sectoral collaborators in both the social-obstetric and social care system in the community.

*“I would not have believed that this girl with mild schizophrenia would succeed in getting her daughter home from the hospital and now I observe normal mother-child interaction when she visits my clinic”* (P18, male GP > 45 years)

### The intangible indicators of vulnerability

The GPs reported cases of pregnant women, whom they perceived to have intangible indicators, not related to any prior known diseases or obvious social problems, evoking their gut feeling of vulnerability. This gut feeling of intangible vulnerability were related to the GPs’ perceptions of abnormal contact or behavior, traits indicating low resilience and a retrospective realization of missed vulnerability.

*Women appearing with an abnormal contact or behavior* could trigger the GPs’ gut feelings’ that something is wrong, and that the pregnant woman might not have the sufficient parent eligibility or might suffer from an undisclosed psychiatric disease or a deviant personality. Especially the GPs were guided by their gut feeling in cases where no prior doctor-patient relation existed, due to the patient was new in the clinic. As a female GP said:

“*I had this girl, who was a new patient and came for removal of her anticonception implant which we removed. Something was odd in the contact with her, and it triggered my attention that something was wrong. After a thorough reading through her file, I discovered that her child was forcibly removed from her home by the social authorities a few years ago”* (P8, female GP, > 45 years)

*Traits indicating low resilience* were related to the individual woman or her available network. Although the GPs’ did not use the term resilience, they mentioned several individual traits of pregnant women evoking their gut feeling of intangible vulnerability. These individual traits was low threshold of stress, coping problems with being pregnant, having ambivalence of the pregnancy and relationship challenges when having a baby. However, there was a gradual transition to normal challenges of motherhood and parenthood as the perinatal period could be considered a vulnerable period.

Often the GPs perceived these women as having low selfcare from lacking the ability to pay attention to their own needs and emotions. This were perceived a frequent challenge for the higher social class women, having high expectations not meeting reality, and these couples were often lacking the ability to seek and accept support from family or professionals.

*“They were this sharp looking couple driving an Audi and carrying designer sunglasses – completely streamlined upper class people you know. It was late in her pregnancy when she first caught my attention, as it appeared how horrible she felt, and that they simply could not embrace the changes that were awaiting them. It was awfully hard for them to accept help we offered”* (P15, female GP < 45 years)

A history taking indicating poor network with loneliness or missing support from spouse evoked the GPs gut-feeling of intangible vulnerability. Contrary, a history indicating an available network of social support were perceived to increase these women’s chance of having a healthy pregnancy and motherhood. As a female GP stated:

“*These [vulnerable women] might not have a proper network to support them, though we also have cases of vulnerable women where parents are available to support them, which may be their saving”.* (P20, female GP>45 years)

*A retrospective realization of missed vulnerability:* Interestingly, the GPs described being guided by the woman’s appearance in their evaluation of the patient’s resources and risks of vulnerability. If a woman appeared with normal interpersonal behavior and well dressed, they were less likely to elaborate on skills of resilience, and these women could go undetected until developing obvious signs of depression. As a female GP said.

“*I had a pregnant woman where everything in her patient file looked fine, but when she came for the five weeks examination of her child I discovered that there was no eye contact with the baby, and subsequently I realized that the mother had a severe postpartum depression – and I thought ‘why didn’t I discover that?’”* (P1, female GP > 45 years)

Debates among GPs during interviews led some GPs to realize how they often recognized this intangible vulnerability retrospectively. Some GPs told how their gut feelings of intangible vulnerability were evoked years later, when the women or couples presented with frequent child consultations for minor things, such as simple colds, due to their insecurity in judging the child’s needs.

“*I have mothers who are well educated and with strong resources but cannot cope with the task of parenthood. They are so focused on themselves and training and everything must be perfect. They contact me all the time about minor things as they cannot judge the needs of their own child(..) I think that represents a vulnerability(..) So next time she gets pregnant I would definitely categorize her as vulnerable”* (P16, female GP, < 45 years)

Especially challenging situations was when pregnant women was new affiliated in the clinic and no doctor-patient relation existed. A GP told how she was misguided by a woman’s physical appearance.

*“They can easily trick you. I remember a woman, very nicely dressed, coming for her first antenatal consultations. She was a new patient and I had no previous medical record on her. We went through her pregnancy record nice and easy, I asked about alcohol use and she said there was none. At the end of the visit she asked how should go about getting her alcohol treatment transferred to the local alcohol rehab center. She was not currently using alcohol, and therefore had answered no to all the questions. It’s hard when we don’t know them. Based on her appearance I had no idea that she was in alcohol rehab”* (P10, female GP, < 45 years)

## Discussion

### Statement of principal findings

The GPs perceived vulnerability in pregnancy as the net result of psychosocial stressors and available individual resources and social supportive network. They classified vulnerability according to its obviousness. Obvious indicators of vulnerability were known complex social problems, mental health problems including history of abuse and severe somatic comorbidity. Some pregnant women were perceived with intangible indicators, evoking the GPs’ gut feeling of vulnerability- where low resilience was prominent. Pregnant women with seemingly normal socioeconomic resources could delay the identification of vulnerability. This was especially the challenge when there was no pre-existing doctor-patient relation.

### Strengths and weaknesses of the study

Strengths of this study were the semi-structured qualitative approach using focus group interviews and using COREQ criteria to ensure transparency [29]. The choice of using focus groups with a flexible interview guide and open questions welcoming clinical examples encouraged a strong dialogue and provided a deep insight into the GPs’ perceptions of the subject. LBV performed the interviews with a neutral and open mind and probed for clarification and depth of the discussion, which ensured that all participants’ voices were heard. Adjustments were made on the interview guide during the ongoing interview phase, ensuring coverage of all emerged perceptions of vulnerability. Another strength is that the study sought to reach a high information power [32] by continuing with interviews until reaching a study sample large enough to reach answer the research questions.

The research group possessed experience from years of working with antenatal care in general practice and from collaborating with doctors and midwifes in the social-obstetric outpatient clinic. This empirical clinical perspective was a strength as it gave a comprehensive insight into the working environment and possible challenges GPs face when defining the concept of vulnerability in pregnancy. Though, we acknowledge that our experience and preconceptions might have affected the generation and interpretation of the qualitative data, and that addition of other professional expertise might have found other perspectives of what indicates vulnerability in pregnancy.

The use of convenience sampling due to slow recruitment, could be a weakness of this study. Only GPs from partnership practices participated, and these might have been GPs with a natural interest in the topic, which could limit the transferability of the findings. Though it was not an inclusion criterion, the recruited GPs varied broadly in their experience with conducting pregnancy consultations, and represented many different perceptions of indicators of vulnerability in pregnancy. This indicates that we recruited GPs with varying interest in the field of antenatal care. Moreover, it is a strength that we achieved diversity regarding seniority, gender, and practice location from urban, semi-urban and rural areas representing patients from different socioeconomic layers. The GPs in the study represented many different perceptions of indicators of vulnerability in pregnancy, and therefore, we believe that our findings could represent the perceptions of GPs from other Danish regions.

### Findings in relation to other studies

This study adds knowledge to a sparsely covered area, about GPs’ perceived indicators of vulnerability in pregnancy. Previous studies focused on GPs’ perceptions of perinatal mental health problems, where vulnerability was classified as a risk factor of perinatal mental health problems. However, GPs’ understandings of indicators of vulnerability were not explored [24–26, 33, 34].

Studies have shown that GPs are more aware of postpartum depression, and that perinatal depression is underdiagnosed by GPs, with up to 50% missed cases [25, 26, 33]. Studies comparing health care professionals found that health visitors were more knowledgeable and aware of perinatal depression than GPs and midwifes [33], and that midwifes lacked the necessary knowledge, skills and confidence to provide mental health care to pregnant women [35]. Contrarily, our study differed in that it focused on GPs’ perceptions of vulnerability and revealed that GPs had a broad clinical perception of indicators of vulnerability.

This study’s findings on GPs’ perceptions of obvious indicators of vulnerability in pregnancy is supported by *the biopsychosocial model* by Engel and the concept of *organismic thinking* by McWhinney. Both concepts from Engel and McWhinney stresses how illness develops through complex interactions of biological, psychological, and social factors. Coexisting social or psychological factors may trigger development of illness, such as depression, with or without biological genetic predispositions of depression. In this way the biopsychosocial model may explain the obvious and intangible indictors of vulnerability in pregnancy as a precursor for complications in pregnancy, birth, and childhood.

The findings that GPs distinguishes vulnerability according to their apparent severity is in line with other findings from nursing literature, where severe vulnerability is defined as the combination of compromised capacity for self-protection while being dependent on health support to prevent development or deterioration of illness [36].

The GPs’ perceptions of intangible indicators of vulnerability covering traits of low resilience, is supported by theories of Family resilience by Walsh [12]. Walsh defines family resilience as “*the capacity of the family as a functional system, to withstand and rebound from stressful life challenges”*. These capacities involves many key processes—such as making meaning of adversity, having a positive outlook, cooperative parenting, help seeking behavior and communication solving. Similar, the intangible indicators of vulnerability in pregnancy, such as coping problems and relationship challenges in pregnancy and reluctancy to seek help can express low resilience.

The finding of GPs’ having gut feelings indicating an intangible vulnerability in a pregnant woman is in line with findings from other studies, which described gut feelings among GPs as a sense of alarm that somethings is wrong, even if no objective argument was present [37–39]. In these studies, the GPs’ used their gut feeling as a compass in uncertain situations and trusted this feeling to guide them in their decision making. However, our study points that GPs’ were reluctant in trusting their gut feelings when a doctor-patient relation was absent, and the women was judged with normal resources from their visual appearances. This could explain why some women with intangible vulnerability were recognized retrospectively.

## Conclusion

This study has demonstrated how GPs perceive indicators of vulnerability in pregnant women according to their apparent severity and obviousness. Obvious indicators of vulnerability in pregnancy were previously known social determinants of health, psychiatric diseases, or chronic somatic diseases, where a psychosocial etiology was the most dominant framework. The GPs described intangible indicators of vulnerability such as odd contact raising suspicion of undetected psychiatric disease, and signs of low resilience in the women or her environment. However, intangible indicators were also recognized retrospectively in women appearing with normal resources, especially when the GP had no relation to the patient. If the GPs were more aware on their gut feelings of intangible indicators for vulnerability, it would elicit the need for a sound history taking of the pregnant woman’s social network and skills of resilience. As a consequence, this might increase the GPs’ awareness on the pregnant woman’s risk of developing perinatal depression.

### Practice implications and future research

Further evidence is needed on the challenges GPs experience when assessing and addressing indicators of vulnerability in pregnant women, and when managing their care through engagement in cross-sectoral collaboration. Changes might be needed in the organization and structure of antenatal care in general practice and in the cross-sectoral antenatal care collaboration to ensure that proper attention is paid to women with intangible vulnerability.

## Supplementary Information

**Additional file 1**

**Additional file 2**

## Data Availability

The dataset generated and analyzed during the current study are not publicly available due to them containing information that could compromise research participant consent but are available from the corresponding author on reasonable request. Data supporting the findings of this study was used under a license granted specifically for the current study and therefore is not publicly available according to the data protection regulations of Danish Data Protection Agency
