# Supplementary material for: General practitioners’ perceived indicators of vulnerability in pregnancy- A qualitative interview study
Source: BMC Fam Pract. 2021 Jun 26;22:135. doi: 10.1186/s12875-021-01439-3 (PMC8236135; doi:10.1186/s12875-021-01439-3)
Supplement: Supplementary file 1 — Additional file 1 [file 12875_2021_1439_MOESM1_ESM.pdf]

COREQ items_ to reply

Domain 1: Research team and reflexivity

Personal Characteristics

1. Interviewer/facilitator: *The first author LBV conducted the interviews with DEJ as an experienced moderator*
2. Credentials: *The main author LBV is a GP and Ph.D. student at the Research Unit of General Medicine at University of Southern Denmark. DEJ and JS are part time GPs and professors at the Research Unit of General Medicine at University of Southern Denmark. RE is a part time GP and associate professor at the research Unit of general medicine, University of Copenhagen. LIB is a survey expert and associate professor at the Research Unit of General Medicine at University of Southern Denmark.*
3. Occupation: *LBV work as a part time GP and part time Ph.D. student*
4. Gender: *LBV is female*
5. Experience and training: *LBV have attended Ph.D. courses in qualitative study designs and writing qualitative articles. DEJ, JS and RE are all senior researchers with experience of qualitative research traditions.*

Relationship with participants

6. Relationship established: *The first interview was a pilot with GPs (n=5) working as part time researchers in our research unit, and therefore had a prior knowledge to the researcher team.*
7. Participant knowledge of the interviewer: *Prior to interviews the interviewer presented the study aim but without presenting our prior experiences and pre-assumptions in the field*
8. Interviewer characteristics: *The interviewer acted friendly, and like-minded among participants. The interview guide provided a flexible frame with open-ended questions about the GPs' perceptions of what indicates vulnerability in a pregnant woman and welcoming clinical examples. Ongoing adjustments of the interview guide were made to elaborate on new perceptions.* Domain 2: study design

Theoretical framework

9. Methodological orientation and theory: *We chose the qualitative methodology to explore GPs perceived indicators of vulnerability in pregnancy. A qualitative design enabled us to explore the GPs understandings of and perceptions of indicators of vulnerability in pregnancy in the terms of "what, why and how". The safe environment during interview encouraged them to disclose situations of deficient performances when identifying vulnerable pregnant women, and the dialogue rendered the GPs to reflect on their own practices.*
   We applied a pragmatic clinical empirical approach not driven by prior established theoretical framework. However, recognizing that our stance is always affected by theory, during process of analysis we searched for theories to support our data interpretation. We chose the biopsychosocial model by Engel(26) and organismic thinking by McWhinney(27) as a backdrop or inspiration.

Participant selection

10. Sampling*: The study aimed to recruit a purposive sample of GPs with respect to; gender, years of experience, practice type and various practice areas throughout the Region of Southern Denmark representing communities of all socio-economic layers.*
11. Method of approach: *Respondents were recruited via letter, telephone, e-mail and snowball sampling.*
12. Sample size: *Due to slow recruitment, the end sample consisted of a convenience sample of twenty GPs representing only partnership practices*
13. Non-participation: *Almost 60 GPs were contacted, and main reasons for decline was a high workload.*

Setting

14. Setting of data collection: *The interviews lasted approximately 60 minutes and took place at the research unit of general practice in Odense or in the local practice area of participating GPs.*
15. Presence of non-participants: *No other besides participants, the interviewer LBV and moderator DEJ was present*
16. Description of sample: *A heterogeneous sample of GPs with respect to gender (12 females, 8 males), experience with both GP trainees (n= 3), 1-10 years of experience (n=7), 11 years and above experience (n=10), from urban (n=5), semi-urban (n=11) and rural areas (n=4).*

Data collection

17. Interview guide: *The interview guide provided a flexible frame with open-ended questions about the GPs' perceptions of what indicates vulnerability in a pregnant woman and welcoming clinical examples. Ongoing adjustments of the interview guide were made to elaborate on new perceptions.*
18. Repeat interviews: *five focus group discussion were made, which included the pilot test*
19. Audio/visual recording: *All interviews were audio recorded and transcribed verbatim by*
20. Field notes: *the author made field notes after almost all interviews about the characteristics of the interview*
21. Duration: *each interview lasted approximately 60 minutes*
22. Data saturation: *data saturation was discussed among authors*
23. Transcripts returned: *No transcripts were returned to participants for comments or corrections*

Domain 3: analysis and findings

Data analysis

24. Number of data coders: *Three of the authors (LBV, RE and DEJ) read the first two interviews. After the themes were discussed among the authors, LBV conducted the initial coding. The following stepwise analysis was conducted by LBV in cooperation with RE. The research team discussed and reflected on the findings until consensus was reached.*
25. Description of the coding tree: *The coding tree is illustrated in figure 1.*
26. Derivation of themes: *The themes obvious vulnerable pregnant women and intangible vulnerable pregnant women were identified from the data*
27. Software: *NVivo pro version 12 was used to organize the data*
28. Participant checking: *As no transcripts was returned to participants, no participants provided feedback.*

Reporting

29. Quotations presented: *quotations were presented to illustrate themes, identified by participant number.*
30. Data and findings consistent: *Consistency were found between the data and the findings, as the findings were recontextualized against the origin interview material.*
31. Clarity of major themes: *The major themes of participants characterizing indicators of vulnerability in pregnant women in levels according to their obviousness were clearly presented in the findings.*
32. Clarity of minor themes: *categories of minor themes were presented in the text and figures.*
